# Genome-Wide In Silico Analysis of Leucine-Rich Repeat *R*-Genes in *Perilla citriodora*: Classification and Expression Insights

**DOI:** 10.3390/genes16020200

**Published:** 2025-02-06

**Authors:** Seon-Hwa Bae, Yedomon Ange Bovys Zoclanclounon, Gyu-Hwang Park, Jun-Dae Lee, Tae-Ho Kim

**Affiliations:** 1 Fruit Research Division, National Institute of Horticultural and Herbal Science, Rural Development Administration, Iseo-myeon, Wanju-gun 55365, Republic of Korea; bae209@korea.kr; 2 Plant Sciences and the Bioeconomy, Rothamsted Research, Harpenden AL5 2JQ, UK; ange.zoclanclounon@rothamsted.ac.uk; 3 Genomics Division, National Institute of Agricultural Sciences, Rural Development Administration, Jeonju-si 54874, Republic of Korea; guhwang01@korea.kr; 4 Department of Horticulture, College of Agriculture and Life Sciences, Jeonbuk National University, Jeonju-si 54896, Republic of Korea

**Keywords:** disease resistance, nucleotide-binding site leucine-rich repeat (NBS-LRR), resistance genes (*R*-genes), genomic distribution, tissue-specific expression, *Perilla citriodora* genome

## Abstract

Background: Resistance (*R*) genes are crucial for defending Perilla against pathogens like anthracnose, downy mildew, and phytophthora blight. Nucleotide-binding site leucine-rich repeat (NBS-LRR) genes, the largest *R*-gene family, play a central role in immunity. This study aimed to identify and characterize NBS-LRR genes in *P. citriodora* ‘Jeju17’. Methods: Previously conducted genome-wide data for ‘Jeju17’ were analyzed in silico to identify NBS-LRR genes. Results: A total of 535 NBS-LRR genes were identified, with clusters on chromosomes 2, 4, and 10. A unique RPW8-type *R*-gene was located on chromosome 7. Conclusions: This study provides insights into the NBS-LRR gene family in ‘Je-ju17’, highlighting its role in disease resistance and evolutionary dynamics. By identifying can-didate *R*-genes, this research supports breeding programs to develop disease-resistant cultivars and improves our understanding of plant immunity.

## 1. Introduction

Plants have developed a variety of defense systems to protect themselves from environmental pathogens and plant pests [1,2]. A critical component of these systems is plant disease resistance genes (*R*-genes), which play a pivotal role in recognizing specific pathogens and triggering defense responses, including localized cell death and systemic acquired resistance [3,4,5]. Among these, a significant majority (approximately 80%) contains a central nucleotide-binding site leucine-rich repeat (NBS-LRR) domain, and these are therefore termed NBS-LRR genes [3]. These pathways confer resistance against a broad spectrum of pathogens, including fungi, bacteria, insects, nematodes, and viruses [6,7,8,9,10].

Numerous studies have demonstrated the protective functions of *R*-genes in various crops, including Arabidopsis thaliana [11], rice (*Oryza sativa*) [12,13,14], wheat (*Triticum aestivum*) [15,16], potato (*Solanum tuberosum*) [17], cotton (*Gossypium* spp.) [18], Brassicaceae species [3], sunflower (*Helianthus annuus*) [19], and mint (*Mentha* spp.) [20]. To date, more than 300 resistance genes have been successfully cloned, emphasizing their critical role in plant defense. A comprehensive understanding of the full repertoire of *R*-genes, particularly NBS-LRR genes, is essential for uncovering their evolutionary significance, identifying functional *R*-genes, and developing resistant plant materials to enhance crop protection [6,7,21]. Recent advancements in genomic technologies have facilitated the genome-wide identification and characterization of NBS-LRR genes across various plant species. For instance, studies in Solanum species have revealed that NBS-LRR genes are predominantly located at chromosomal termini, suggesting a role in rapid adaptation to evolving pathogens [22]. Similarly, the expansion of NBS-LRR genes in plant genomes has been associated with whole-genome duplication events, which contribute to the diversification of plant immune responses [23]. Furthermore, the expression of NBS-LRR genes is regulated by microRNAs, which fine-tune their activity in response to pathogen attacks, highlighting the complexity of plant immune regulation [24].

These NBS-LRR proteins recognize effectors secreted by pathogens either directly or indirectly and subsequently activate downstream signaling pathways, leading to the activation of plant defense responses against various classes of pathogens including fungi, bacteria, insects, nematodes, and viruses [10]. The protein structure of NBS-LRR is composed of three parts: the N-terminal domain for downstream signaling, the central NB-ARC domain of the signal transduction ATPase domain (STAND) family for regulating *R*-gene activity, and the carboxyl-terminal (C-terminal) LRR domain(s) for pathogen recognition specificity and possibly additional downstream signaling [25,26,27].

*R*-genes play a significant role in the evolutionary arms race between plants and pathogens in the immune system of plants [21]. Here, we focus on the *R*-genes containing the NBS domain, which are among the most frequently cloned and characterized genes in plants [8,22]. In plants, *R*-genes are categorized into two groups: intercellular NBS-LRRs and extracellular receptor-like proteins (RLPs), with the vast majority being NBS-LRRs [28]. NBS-LRRs are subdivided into two main types based on the -terminal domain present: NBS-LRRs with the toll and interleukin-1 receptor (TIR) domain (TN) and NBS-LRRs with the coiled-coil domain (CNL) [6,29]. Additionally, several studies have identified a small group of resistance to powdery mildew 8 (RPW8) genes, known as ancient distinct members of the NBS-LRR gene family closely related to CNL [30,31,32,33,34].

This study aimed to characterize the diploid ‘Jeju17’ genome by focusing on *R*-genes. Specifically, we identified, classified, and analyzed the genomic distribution and differential expression patterns of *R*-genes across various organs through in silico analysis. The findings from this study provide a foundational understanding of the NBS-LRR gene family in *P. citriodora*, offering valuable insights for future molecular breeding programs to enhance disease resistance.

## 2. Materials and Methods

### 2.1. Identification of P. citriodora ‘Jeju17’ NBS-LRR Genes and Classification

This study utilized previously published genomic resources [35] to access the genome and annotation data of *P. citriodora* ‘Jeju17’, which provided the foundational protein sequences and necessary annotation details for identifying and analyzing the NBS-LRR gene family in this cultivar. Protein domains were obtained from the genome assembly using Pfam v.33.1 [36]. A pipeline, including BLAST and Hidden Markov Model (HMM) analysis (http://hmmer.org, accessed on 26 November 2020) [7], was used to identify nucleotide-binding and leucine-rich repeat (NLR) genes in *P. citriodora*. The search involved BLASTp and HMM search programs, employing an e-value of 10^−5^, and scanning for the conserved NBS domain using the HMM scan program against the Pfam database (http://pfam.xfam.org, accessed on 26 November 2020). The classification of NLR genes was based on the Pfam accession number of NB_ARC (PF00931), and CC domain identification utilized a motif-based approach with the NLR-Annotator tool [37]. Identified NLR genes’ conserved motifs were examined using MEME suite 5.3.0 (https://meme-suite.org, accessed on 26 November 2020), set to a maximum of 20 motifs in accordance with [38].

### 2.2. Chromosomal Localization, Gene Density, and Phylogenetic Analysis of NBS-LRR Genes

The chromosomal locations of NLR genes were determined using the genome sequence dataset of ’Jeju17’. Gene density per chromosome was calculated using a 100 kbp window. Both the location and density of NLR genes within the ‘Jeju17’ genome were visualized using the R-language-based package RIdeogram 0.2.2 [39].

Prior to phylogenetic construction, multiple sequence alignment was performed using MAFFT 7.475 [40]. The alignment was then trimmed with trimal 1.4.4 [41] to exclude poorly aligned regions. A maximum likelihood phylogenetic tree was constructed with IQ-TREE 2.0.3 [42], employing ultrafast bootstrap with 1000 replicates following the model predicted by the embedded package Modelfinder version 1.4.2 [43]. The phylogenetic tree was visualized online using Evolview 3.0 [44].

### 2.3. NLR Gene Synteny and Duplication Investigation

To identify putative NLR gene synteny and duplication in ‘Jeju17’, an all-by-all alignment of protein files was followed by the use of MCScanX 2.0 [45], a synteny and duplicate gene classifier program running with default settings.

### 2.4. Differentially Expressed NLR Gene Analysis

In our previous study, RNA sequencing and genome annotation for *P. citriodora* ‘Jeju17’ were conducted, providing the foundational dataset utilized in the current analysis [35]. Specifically, RNA datasets were collected from buds, leaves, and seeds, employing three biological replicates per organ. The Illumina 151 bp length paired-end data were screened for adapter contamination and preprocessed using the fastp tool 0.20.1 [46] with default settings. The clean RNA datasets were mapped back to the genome using HISAT2 2.2.1 [47], and abundance estimation was performed using featureCounts 2.0.1, followed by the DESeq2 1.30.0 pipeline [48] for differential expression analysis under the R 4.0.3 environment [49].

## 3. Results

### 3.1. Identification and Classification of ‘Jeju17’ R-Genes

**Figure 1:**
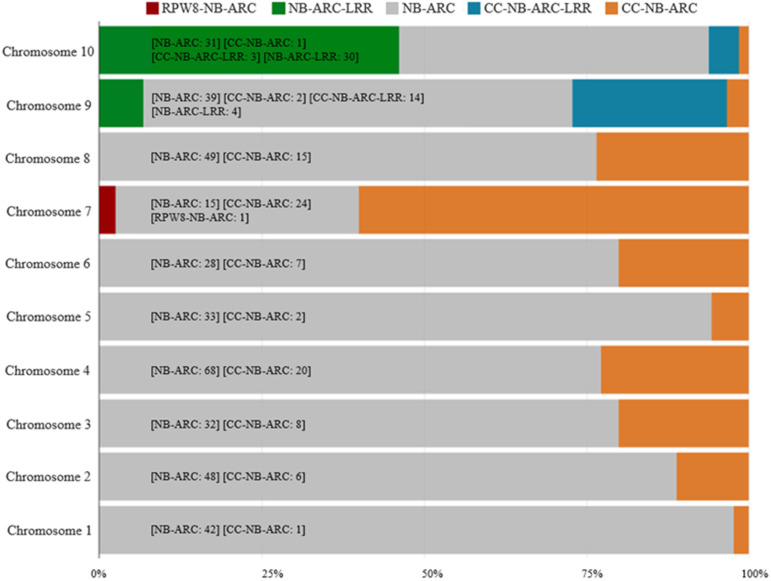
The distribution of NB-ARC genes across the ‘Jeju17’ genome. The colors depicted in the graph represent the various *R*-gene types. The numbers reflect the count of genes identified for each *R*-gene type.

A total of 535 NBS-LRR protein coding sequences (CDSs) were identified in ‘Jeju17’ (Table 1). Based on their protein domain combinations, these genes were categorized into five classes (Table 1). Additionally, all NBS-LRR genes in ‘Jeju17’ contain the NBS domain, 104 genes exhibit the CC domain, and only one contains the RPW8 domain. Using the previously described method, the identification of *R*-genes in ‘Jeju17’ resulted in 535 resistance genes (Table 1), representing 1.63% of the 32,769 ‘Jeju17’ genes. Based on their protein domain combinations, these *R*-genes were classified into five groups, resulting in 395 NB-ARC, 87 CC-NB-ARC, 35 NB-ARC-LRR, 17 CC-NB-ARC-LRR, and 1 RPW8-NB-ARC type (Table 1). For the RPW8-NB-ARC-type gene, only one gene was located on chromosome seven (Figure 1).

### 3.2. NLR Gene Distribution and Phylogenetic Tree Construction in the ‘Jeju17’ Genome

**Figure 2:**
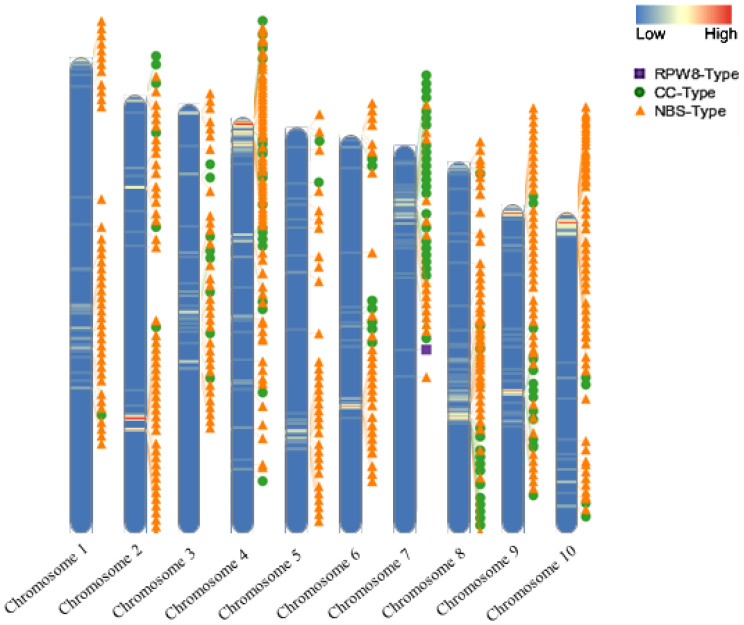
The chromosomal distribution of the *R*-genes in ‘Jeju17’. The heatmap illustrates gene density and *R*-genes along with their type labels.

The distribution of *R*-genes in the ‘Jeju17’ genome was mapped along the ten chromosomes using a window size of 100,000 bp and position information from the annotation file. A total of 220,000 loci, exclusively comprising the NB-ARC gene type, were identified according to their physical positions. The three main types of *R*-genes identified in this study—NBS-type, CC-type, and RPW8-type—were distributed differentially along the chromosomes (Figure 2). NBS-type genes (orange triangles) were the most abundant, forming clusters along chromosomes 2, 4, and 10, while CC-type genes (green circles) were relatively more dispersed. Chromosome 7 was unique in that it contained 22 *R*-genes, the highest number of clusters, and also housed the RPW8-type gene (purple square) as a singleton, further emphasizing its distinctiveness within the genome. The distribution of NBS-LRR genes across the ten chromosomes was uneven, with no significant correlation detected between chromosomal length and the number of NBS-LRR genes. These findings highlight the clustered organization of *R*-genes, particularly on chromosomes 2, 4, 10, and 7, and underscore the unique role of the RPW8 gene in plant immunity.

**Figure 3:**
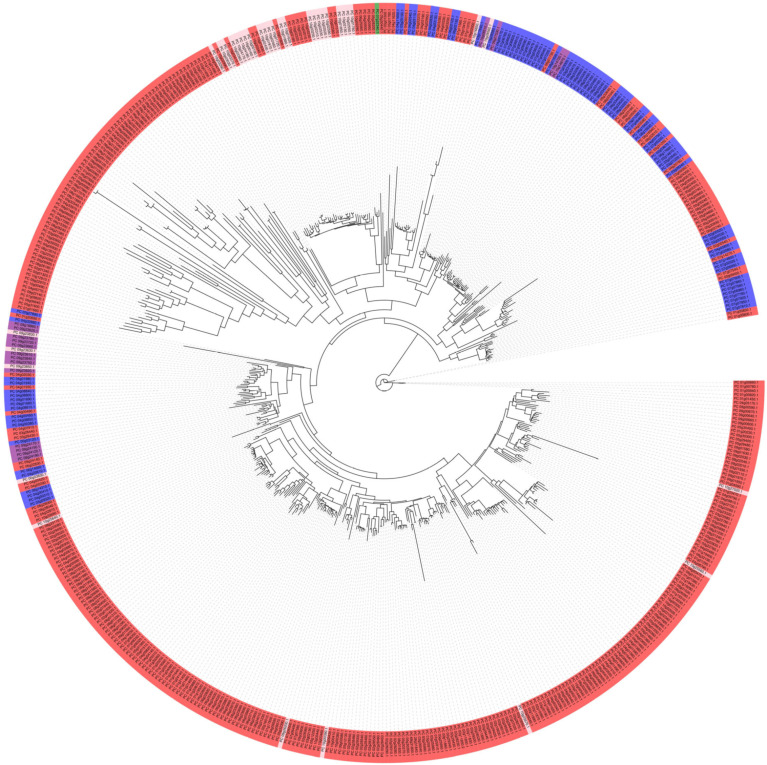
The phylogenetic reconstruction of NBS-LRR proteins in ‘Jeju17’. The clades are color-coded: NB-ARC in red, CC-NB-ARC in blue, CC-NB-ARC-LRR in purple, and NB-ARC-LRR in pink, with the tree, while the unique RPW8 type (green) is isolated in a single node.

A phylogenetic tree was constructed using the 535 identified resistance genes based on a maximum likelihood estimation scheme (Figure 3). The most representative NB-ARC types (red color) were notably clustered together. The CC types appeared along some branches of the tree, while the unique RPW8 type (green) was isolated in a single node, providing a valuable resource for understanding the functional activation of this gene.

### 3.3. Expression Analysis of R-Genes in Tissues (Bud, Leaf, and Seed)

**Figure 4:**
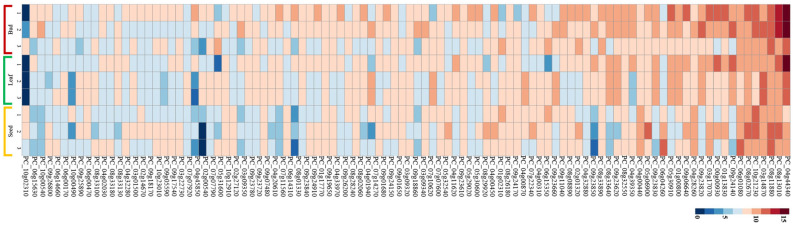
Expression analysis of *R*-genes from ‘Jeju17’ visualized as heatmap. This heatmap was generated using RNA-seq normalized data for ‘Jeju17’ *R*-gene expression in different tissues (bud, leaf, and seed).

The present study investigated the expression patterns of *R*-genes in ‘Jeju17’ tissues, including bud, leaf, and seed tissues. Globally, *R*-genes were less expressed in all tissues (Figure 4). Among the analyzed genes, PC_04g44340 exhibited the highest expression levels, with particularly strong expression in both bud and leaf tissues. This gene showed significantly higher expression levels in leaf tissues, indicating a tissue-specific expression pattern. In addition, PC_08g13010 also displayed relatively high expression levels in bud and leaf tissues, though its expression levels were lower compared to PC_04g44340. In leaf tissues, a total of five highly expressed genes were identified, including PC_04g44340, PC_03g14870, PC_08g13010, PC_09g24140, and PC_00g00930 (Figure 4). In seed tissues, seven highly expressed genes were identified, namely PC_08g13010, PC_08g11880, PC_10g1021230, PC_08g02670, PC_06g01080, PC_06G04260, and PC_05g06900. In contrast, most *R*-genes exhibited minimal expression levels in seed tissues, with no distinctively high-expression genes identified apart from those listed. These results demonstrate the variation in *R*-gene expression across tissues, with a notable emphasis on leaf and bud tissues, where certain genes such as PC_04g44340 and PC_08g13010 are prominently expressed. This highlights the potential tissue-specific regulation of *R*-genes in ‘Jeju17’. Additionally, a total of five (PC_04g44340, PC_03g14870, PC_08g13010, PC_09g24140, PC_00g00930) and seven (PC_08g13010, PC_08g11880, PC_10g1021230, PC_08g02670, PC_06g01080, PC_06G04260, PC_05g06900) highly expressed genes were identified in leaf and seed tissues, respectively (Figure 4).

**Figure 5:**
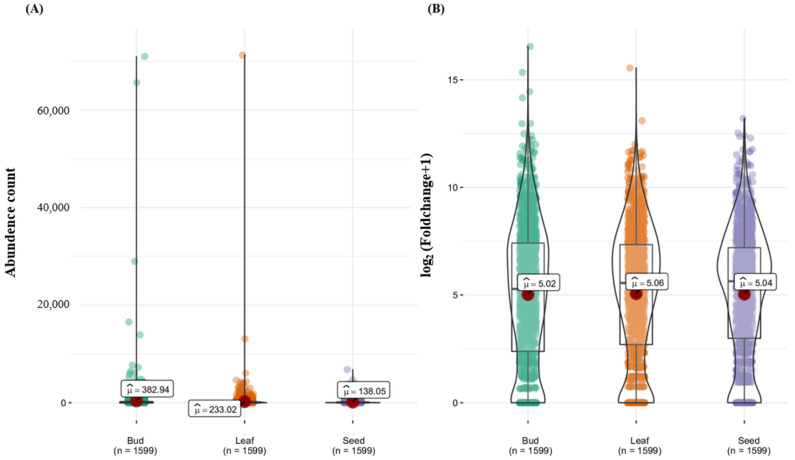
A comparison of the mean number of *R*-genes between tissues (bud, leaf, and seed). (**A**) The *x*-axis represents the average number of *R*-genes by tissue (bud, leaf, and seed). (**B**) The *x*-axis represents the average log_2_ value of *R*-genes by tissue (bud, leaf, and seed).

The average *R*-gene comparison among tissues showed mean gene counts of 382.94, 233.02, and 138.05 for bud, leaf, and seed tissues, respectively (Figure 5A). No significant difference was observed in gene count among the three tissues (Figure 5B). The top 15 (≥1000 gene counts) *R*-genes were predominantly expressed in bud tissues (Figure 4 and Figure 5). In particular, the highly expressed gene identified is pleiotropic drug resistance, belonging to the subfamily of ABC transporters, which are adenosine triphosphate (ATP)-binding cassettes (Figure 5A).

Overall, the present study identified a set of 535 *R*-genes from the ‘Jeju17’ genome. The genomic distribution, expression level, and classification were comprehensively described. These preliminary findings may pave the way for the molecular breeding of ‘Jeju17’ for disease resistance.

## 4. Discussion

### 4.1. Diversity and Evolution of R-Genes in ‘Jeju17’

*R*-genes play a critical role in plant defense by recognizing various pathogens and pests and activating immune responses. In ‘Jeju17,’ all genes exhibit the NBS domain, surpassing the oil crop sesame, which possesses 171 *R*-genes [50], and olives, which contain 459 *R*-genes [51]. Notably, no TIR type was found among the identified *R*-genes. Although the TIR domain’s absence is common in monocots but rare in eudicots [52], exceptions have been reported, such as sugar beet [53]. The lack of the TIR domain in the members of Lamiales might necessitate further evolutionary studies. Moreover, the TIR domain’s presence is associated with NRG1 proteins for activating the TIR signal transduction pathway [54,55]. The absence of the TIR domain could indicate lineage-specific adaptations in Lamiales, suggesting unique evolutionary pressures that shaped the immune system of this clade. Further phylogenomic analyses focusing on TIR domain evolution across dicot species could provide deeper insights into these adaptations.

### 4.2. Chromosomal Distribution and Defense Mechanisms of NBS-LRR Genes

The distribution of NBS-LRR genes in ‘Jeju17’ reveals their uneven localization across the 10 chromosomes, with a clear tendency to form high-density clusters at chromosomal termini. These patterns align with findings in other plant species, where clustering is associated with enhanced defense mechanisms, enabling plants to respond rapidly and locally to biotic stresses [56]. High-density clusters of NBS-LRR genes were identified on chromosomes 2, 4, and 10, serving as key loci for plant immunity by harboring genes critical for recognizing pathogen effectors and activating downstream defense pathways. NBS-LRR genes are known to play a pivotal role in recognizing pathogen-derived effectors and initiating downstream signaling pathways [57]. This triggers the activation of localized cell death and systemic acquired resistance, providing robust protection against fungal, bacterial, viral, nematode, and insect pathogens [8]. These high-density clusters may also play a role in genomic plasticity by facilitating recombination events, which drive the diversification of resistance genes [58]. Similar mechanisms have been observed in rice and wheat, where genomic rearrangements in clustered *R*-genes contribute to rapid adaptation against evolving pathogens [59].

### 4.3. Functional Importance and Uniqueness of RPW8 Gene

In particular, chromosome 7 harbors the RPW8-type gene, a singleton with broad-spectrum pathogen resistance, highlighting its potential functional specialization [30,60]. The RPW8 gene is known to manifest in the early growth stages of land plants and has been documented to confer resistance to powdery mildew fungi [60]. Downy mildew, prevalent in *Perilla* seeds, shares common characteristics with fungal diseases such as powdery mildew. As for the specifically identified RPW8 gene, it was reported that one RPW8 gene was also found in *Mimulus guttatus* of the Lamiaceae family, demonstrating similar results to those in ’Jeju17’ [61], making it particularly intriguing to explore the maintenance and evolutionary history of this gene, which mediates broad-spectrum pathogen resistance and was initially discovered in the protein encoded by the polymorphic RPW8 locus [30,62]. RPW8 genes have also been identified in *Castanea mollissima* and *Mimulus guttatus*, with counts of 15 and 1, respectively [61,62,63]. Future studies could explore the functional versatility of RPW8 in crops by using CRISPR/Cas9-mediated gene editing to introduce this gene into species lacking broad-spectrum resistance. Transcriptomic analyses under powdery mildew stress could also reveal the dynamic regulation of RPW8 and its downstream signaling pathways [64].

### 4.4. Evolutionary Conservation and Functional Roles of R-Gene Clusters

In general, *R*-gene clusters have been reported in many plant species [7,19,65,66,67], and the clustering patterns observed in ‘Jeju17’ are consistent with those in soybean, rice, and wheat. For example, soybean chromosome 13 hosts clusters associated with resistance to bacterial, fungal, and viral diseases. Rice chromosome 11 contains *R*-genes providing resistance to the brown planthopper (*Nilaparvata lugens*), while wheat chromosome 1 harbors *Lr1* and *Lr10*, critical for leaf rust resistance (*Puccinia triticina*) [7,19,65,66,67]. These patterns facilitate genomic rearrangements, driving the evolution of new resistance genes and highlighting the functional significance of *R*-gene clusters [68,69,70,71]. Specifically, Shao et al. (2014) emphasized the long-term evolution of NBS-LRR genes across various plant families, while McIntosh (1979) provided a catalog of resistance genes in wheat, and Cloutier et al. (2007) identified *Lr1* as a key gene in the psr567 gene family. Richly et al. (2002) discussed the mode of amplification and reorganization of resistance genes during the recent evolution of *Arabidopsis thaliana*, shedding light on the dynamics of gene family evolution in plants [72]. Recent studies in *Capsicum baccatum* also identified NBS-LRR clusters and RPW8-domain-containing genes, emphasizing the evolutionary conservation of these features in plant immunity [7]. The evolutionary conservation of clustering highlights its functional importance in maintaining genomic stability while enabling plants to adapt to diverse biotic stresses. Comparative studies across more species could identify conserved motifs or regulatory elements within these clusters, offering new targets for genetic engineering [73]. 

### 4.5. Breeding Applications of R-Gene Clusters

The clustering patterns in ‘Jeju17’ and other species suggest shared evolutionary pressures shaping the *R*-gene repertoire, underscoring their role in coordinated defense responses. Overall, the clustering of *R*-genes in ‘Jeju17’ highlights their importance in plant defense and offers promising targets for molecular breeding. These examples demonstrate the critical roles of *R*-gene clusters in plant immune responses across various species. The clustering of these genes likely facilitates coordinated defense mechanisms, emphasizing their potential application in breeding programs aimed at improving resistance to biotic stresses. The identified clusters on chromosomes 2, 4, and 10, as well as the RPW8-type gene on chromosome 7, provide valuable opportunities for exploring evolutionary dynamics and enhancing crop resistance through genomic tools. Marker-assisted selection (MAS) and genome-wide association studies (GWASs) could be employed to identify candidate *R*-genes within these clusters, accelerating breeding programs for enhanced stress tolerance [74].

### 4.6. Tissue-Specific Expression and Functional Roles of R-Genes

In this study, our results demonstrated that *R*-genes exhibit differential expression across various tissues, with a notable predominance in bud tissues. Among the highly expressed *R*-genes, CC-NB-ARC types were predominant, as revealed by bioinformatics analysis, suggesting their significant role in localized immune responses. Notably, one of the highly expressed genes is related to pleiotropic drug resistance (PDR) and belongs to the subfamily of ATP-binding cassette (ABC) transporters, which are adenosine triphosphate (ATP)-binding cassettes [75,76,77]. This gene has been found exclusively in fungi and plants and is reported to play critical roles in both abiotic and biotic stress responses. Its high expression levels underscore its potential involvement in stress adaptation and highlight the functional diversity of *R*-genes in ‘Jeju17’. These findings emphasize the importance of tissue-specific *R*-gene regulation in enhancing plant resistance mechanisms. While these insights are derived from bioinformatics analysis, further validation through molecular biological experiments is necessary to confirm these observations and elucidate the precise functional roles of these genes.

## 5. Conclusions

A total of 535 NBS-LRR genes were identified from the diploid *Perilla citriodora* ‘Jeju17’ genome sequence by analyzing their structure and distribution on chromosomes in silico. Characterizing disease resistance genes is essential for understanding how wild *Perilla* defends itself against various pathogens. Notably, this study is the first to identify disease resistance genes in the form of NBS-LRRs in diploid *Perilla*. Comparing resistance genes with those of Lamiaceae plants and analyzing expression and quantification in different *Perilla* tissues allow us to predict the function of resistance genes in diploid *Perilla*. This knowledge, together with resistance genes from tetraploid cultivars of *Perilla*, is crucial for creating an enhanced *Perilla* disease defense information system. Integrating and comparing various diploid *Perilla* disease resistance genes, including those identified in this study, support species conservation and the preservation of genetically diverse habitats. This information on disease resistance genes also plays a vital role in exploring genetic variations over time and changes in species in response to environmental shifts. 

## Figures and Tables

**Table 1:** Classification of nucleotide-binding site leucine-rich repeat (NBS-LRR) genes in ‘Jeju17’.

Species	Class	Type	Number of Genes
‘Jeju17’	1	CC-NB-ARC	87
	2	CC-NB-ARC-LRR	17
	3	NB-ARC	395
	4	NB-ARC-LRR	35
	5	RPW8-NB-ARC	1
	Total		535

## Data Availability

The original contributions presented in the study are included in the article, further inquiries can be directed to the corresponding author.
